# Recurrent Neurotropic Chloroma: Report of a Case and Review of the Literature

**DOI:** 10.1155/2011/854240

**Published:** 2010-12-01

**Authors:** Richard Bakst, Ann Jakubowski, Joachim Yahalom

**Affiliations:** ^1^Department of Radiation Oncology, Memorial Sloan-Kettering Cancer Center, 1275 York Avenue, New York, NY 10065, USA; ^2^Department of Medicine, Memorial Sloan-Kettering Cancer Center, 1275 York Avenue, New York, NY 10065, USA

## Abstract

We are reporting a case of a young woman with acute myelogenous leukemia status postallogeneic transplantation who developed multiply recurrent chloromas occurring along peripheral nerves in the absence of bone marrow relapse, all treated with radiation therapy. The patient is currently free of disease nearly four years after her first posttransplant chloroma. The case presented is unique for its isolated peripheral nervous system involvement, rare posttransplant occurrence, and indolent course without marrow relapse despite multiple extramedullary recurrences.

## 1. Introduction

Chloroma (also known as granulocytic sarcoma or myeloid sarcoma) is a rare, extramedullary tumor composed of immature granulocytic cells. It was first described in 1811 [1] and coined “chloroma” by King [2] in 1853 because of its green color. Its relationship with leukemia was later established in 1893 [3]. 

 Chloromas are reported in 2.5%–9.1% [4–6] of patients with acute myeloid leukemia (AML) and occur concomitantly, following, or rarely antedating the onset of leukemia [7]. The presence of an extramedullary relapse of leukemia is often associated with a poor prognosis [8]. Chloromas' clinical manifestations are diverse given their various sites of occurrence. Involvement of isolated peripheral nerves by chloromas is exceedingly rare and is documented only in limited reports preceding more widespread relapse in the majority of cases [9–12].

## 2. Case Report

In June 2004, a 21-year-old white woman presented with spiking fevers, bruising, shortness of breath, and malaise. Laboratory findings revealed a white blood cell count of 21,700/*μ*L with 47% circulating blasts. Subsequent bone marrow evaluation demonstrated AML with del(9q). Lumbar puncture was negative for cerebral spinal fluid (CSF) involvement. The patient underwent prompt treatment with cytarabine (Ara-C) 200 mg/m^2^ continuous infusion for seven days and idarubicin 12 mg/m^2^ daily for three days. Bone marrow evaluation at day 14 revealed residual leukemia, and repeat bone marrow evaluation three weeks later was negative. The patient subsequently received consolidative treatment with four cycles of high dose Ara-C. 

While receiving the chemotherapy, the patient developed pain in her left shoulder which radiated down the left arm from the elbow into the ulnar two fingers with numbness in those digits. The pain progressed, and further work-up was pursued including electromyography (EMG), which was consistent with an ulnar neuropathy. The patient underwent transposition of the ulnar nerve and carpal tunnel release because of new slight thumb numbness. The patient's symptoms progressed despite the procedure to include impaired dorsiflexion of the left great toe, numbness of her right calf, and numbness and weakness of the right thumb and index finger. Ten months after her initial diagnosis, associated fatigue prompted a repeat bone marrow evaluation, which was consistent with relapsed AML. Magnetic resonance imaging (MRI) of the brachial plexus and lumbar spine revealed bilateral brachial plexus involvement by a presumed chloroma and leukemic infiltration of the left and right lumbosacral plexus.

The patient initiated treatment with mitoxantrone and etoposide. Repeat bone marrow evaluation was negative and repeat MRIs of the brachial plexus and lumbar spine demonstrated resolution of the leukemic involvement. While post-treatment biopsy of the chloroma sites was not performed, it was felt that based on the available imaging, clinical improvement, and bone marrow aspiration results, that the patient had attained a complete response to treatment. The patient underwent an unrelated donor-matched allogeneic bone marrow transplant. The conditioning regimen included total body irradiation (TBI) to 1375 cGy in 11 fractions administered three times daily with a consolidative boost to the left brachial plexus to 600 cGy in four daily fractions. The boost was added because residual microscopic disease could not be ruled out. Graft versus host disease (GVHD) prophylaxis included tacrolimus plus methotrexate. 

Repeat bone marrow aspiration posttransplant demonstrated cytogenetic remission with full donor chimerism and normal karyotype. The patient completed five monthly doses of intrathecal Ara-C (70 mg) as central nervous system (CNS) prophylaxis. Cytopathology on all the CSF samples was negative for malignancy. The patient's posttransplant course was complicated by hepatic fungal infections, GVHD, involving the upper gastrointestinal tract and liver, and renal insufficiency resulting in a very slow but physically remarkable recovery to eventually include triathlon training. While on immune suppression including tacrolimus and prednisone, the patient developed leukemia cutis of the right chest that resolved completely with the tapering of immunosuppressive medications. A bone marrow evaluation at the time of the skin biopsy revealed a normal karyotype, all donor by diagnostic molecular pathology.

Approximately one year posttransplant, the patient developed progressive left intrinsic hand weakness with an associated palpable mass in her left upper extremity. An MRI study was consistent with a chloroma of the biceps brachi along the course of the musculocutaneous nerve. Fine needle aspiration of the mass was consistent with leukemic infiltration; CSF analysis and bone marrow aspiration at the time of the peripheral nerve biopsy were negative for leukemic involvement, and polymorphism analysis with DNA primers demonstrated complete donor engraftment. The patient received a course of radiation therapy to 3000 cGy in 15 fractions to the mass, with complete clinical resolution of the lesion by treatment completion. 

**Figure 1:**
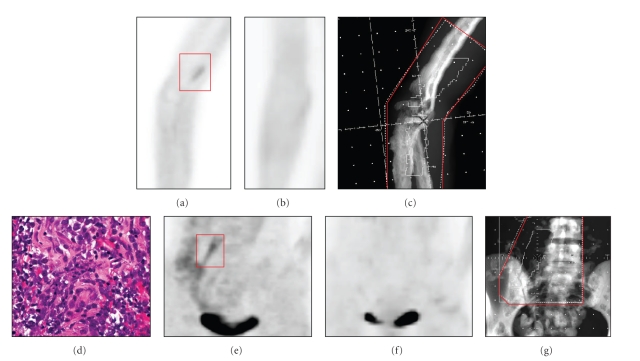
Recurrent neurotropic chloroma of the right ulnar nerve on (a) pretreatment Positron Emission Tomography (PET) outlined in red demonstrating resolution (b) of hypermetabolic activity after treatment to 24 Gy to (c) the field outlined in red. Biopsy proven recurrent neurotropic chloroma on high power view with Heamtoxylin-Eosin staining (d) along the right psoas muscle outlined in red on (e) pretreatment PET demonstrating resolution (f) of hypermetabolic activity after treatment to 24 Gy to (g) the field outlined in red.

Seven months after completion of the patient's last course of radiation, she developed new right arm and leg pain. Work-up including MRIs demonstrated three presumed chloromas near the right elbow (ulnar nerve), right upper deltoid (axillary nerve), and right psoas region (lumbar plexus). Biopsy of the right psoas lesion was consistent with chloroma (Figure 1); CSF analysis and bone marrow biopsy were negative and polymorphism analysis did not detect any host cells. The patient underwent her third course of radiation to the right elbow, right deltoid region, and right paraspinal psoas mass, each to 2400 cGy in 12 fractions. All sites demonstrated a clinical and radiographic response (Figure 1) on followup. Three months after completion of treatment, the patient developed new pain in her left first and second toes. Imaging was consistent with a left peroneal nerve chloroma for which she received 2400 cGy in 12 fractions.

The patient had been completely tapered off immunosuppression and was doing well on no further therapy until 14 months later, when she palpated a new mass in her left forearm, which was biopsied and consistent with a leukemic infiltrate. Further work-up did not reveal any systemic disease with bone marrow evaluation showing full donor chimerism and no CSF involvement. The patient was neurologically asymptomatic; however, neurological examination was consistent with median nerve involvement. A course of 2400 cGy in 12 fractions to the left forearm was initiated with clinical resolution. Despite the patients multiple recurrences and courses of radiation, she remained in excellent physical condition with continued participation in triathlons and road races. 

**Figure 2:**
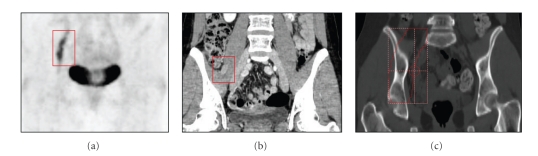
Recurrent neurotropic chloroma of the right femoral nerve outlined in red on (a) pretreatment Positron Emission Tomography (PET) and (b) coronal Computerized Tomography (CT) imaging with the (c) CT-based treatment field outlined in red.

Despite receiving a low dose donor lymphocyte infusion of 1 × 10^5^ CD3+ cells/kg several months before, the patient developed right lower extremity weakness along the femoral distribution approximately one year after her last radiation treatment. MRI, computerized tomography (CT), and positron emission tomography (PET) were consistent with a right femoral nerve chloroma extending into the right inguinal canal (Figure 2). The patient received another course of 2400 cGy in 12 fractions with a cone down to avoid overlap with the prior radiation field (right psoas chloroma). Clinical improvement was observed during the course of treatment without any associated treatment toxicities. Repeat MRI one month after treatment demonstrated complete resolution.

Since completion of her last course of radiation therapy, she has received no further therapy and has not shown any evidence of recurrence of chloroma or leukemia; the patient plans to resume her triathlon training. 

## 3. Discussion

The most common locations of chloromas are skin, soft tissue, bone, periosteum, and lymph nodes [8]. Central nervous system (CNS) involvement is rare but has been described in numerous case reports [13–15]. By contrast, isolated peripheral nervous system (PNS) involvement without epidural or leptomeningeal involvement is exceedingly rare. An extensive review of the literature documents only four such cases [9–12]. In three of the cases, the patient's leukemia subsequently recurred within a short interval either in the CSF or marrow resulting in death; autopsy in the fourth case demonstrated leukemic cells in the eyes but none in the CSF or marrow. 

The incidence of chloromas developing after allogeneic transplantation has been reported in 0.2–1.3% of patients undergoing transplantation with overall poor survival [16, 17]. Interestingly, in these two retrospective series a large portion (48%) of chloromas occurred in the CNS structures and ovaries suggesting that chloromas might arise in sanctuary sites where leukemic cells survive treatment with chemoradiotherapy. The management of chloromas after allogeneic transplantation is controversial. Aggressive treatment with a second transplant is an option; however, its efficacy and safety is not established. Donor lymphocyte infusion has also been attempted with clinical promise in a select few cases [18]. Local therapy with radiation has been utilized for palliation as well [17]. 

In the current case, involvement of the PNS pre- and posttransplant suggests that peripheral nerves serve as a sanctuary site. Leukemic cells in CNS structures and the testes, which have inherent barriers [19, 20], are known to escape systemic therapy. The existence of a blood-nerve barrier could explain how relapse of acute leukemia might originate with leukemic cells that have persisted in peripheral nerves [21]. The administration of intrathecal chemotherapy and the use of a testis boost for males during TBI can overcome these barriers; however, no such method exists for bypassing the blood-nerve barrier. It is therefore surprising that more relapses do not occur in the PNS.

Chloromas following transplantation have conventionally been considered the first manifestation of relapsed systemic disease. However, rare isolated extramedullary relapses of leukemia without concomitant involvement of the marrow after allogenic transplantation have been described [22]. In our patient, the selective involvement of extramedullary sites might be attributable to a differential favorable graft versus leukemia (GVL) effect on the marrow as compared with PNS. This has resulted in excellent disease control with radiation therapy alone. The unusually long survival, indolent course, and absence of marrow relapse in this patient, despite repeated chloromas, have been described previously in rare cases posttransplant [23]. Notable in our patient was the response of the leukemia cutis to withdrawal of immune suppression, suggesting that GVL was adequate to control the disease in the skin, a nonprivileged organ site.

Chloromas' rare incidence, their often misdiagnosis [24], and variable locations have resulted in limited clinical experience, and hence in a lack of consensus treatment guidelines. The role of radiation in their management has typically been palliative as low doses have resulted in excellent disease control and symptom relief. In contrast to older studies that support the use of at least 3000 cGy, our experience with this case and others suggests that 2400 cGy is adequate [25]. Furthermore, despite having undergone TBI and multiple courses of low-dose radiation, this patient's treatment-related toxicity has been minimal, suggesting that in this case, carefully planned reirradiation was safe. The applicability of this type of regimen to similar presentations needs to be considered on a case-by-case basis as all re-irradiation cases require careful scrutiny. While the majority of chloromas will lead to systemic relapse warranting systemic therapy, isolated, indolent extramedullary recurrences might represent a growing minority as allogeneic transplantation continues to improve. Such isolated recurrences may benefit from radiation therapy. 

In conclusion, we believe that this case is an important contribution to our limited understanding of chloromas by providing evidence that peripheral nerves can serve as a sanctuary site for leukemic cells and an origin of chloromas. This challenges the belief that chloromas always precede systemic relapse and suggests an evolving role for radiation therapy in the management of chloromas beyond palliation. Further, more comprehensive studies of chloromas are warranted to determine optimal management.
